# Remote targeted implantation of sound-sensitive biodegradable multi-cavity microparticles with focused ultrasound

**DOI:** 10.1038/s41598-019-46022-0

**Published:** 2019-07-03

**Authors:** Xiaoqian Su, Reju George Thomas, Lakshmi Deepika Bharatula, James J. Kwan

**Affiliations:** School of Chemical and Biomedical Engineering, Nanyang Technological University, Singapore, 637459 Singapore

**Keywords:** Therapeutics, Biomedical engineering

## Abstract

Ultrasound-enhanced drug delivery has shown great promise in providing targeted burst release of drug at the site of the disease. Yet current solid ultrasound-responsive particles are non-degradable with limited potential for drug-loading. Here, we report on an ultrasound-responsive multi-cavity poly(lactic-co-glycolic acid) microparticle (mcPLGA MP) loaded with rhodamine B (RhB) with or without 4′,6-diamidino-2-phenylindole (DAPI) to represent small molecule therapeutics. After exposure to high intensity focused ultrasound (HIFU), these delivery vehicles were remotely implanted into gel and porcine tissue models, where the particles rapidly released their payload within the first day and sustained release for at least seven days. RhB-mcPLGA MPs were implanted with HIFU into and beyond the sub-endothelial space of porcine arteries without observable damage to the artery. HIFU also guided the location of implantation; RhB-mcPLGA MPs were only observed at the focus of the HIFU away from the direction of ultrasound. Once implanted, DAPI co-loaded RhB-mcPLGA MPs released DAPI into the arterial wall, staining the nucleus of the cells. Our work shows the potential for HIFU-guided implantation of drug-loaded particles as a strategy to improve the local and sustained delivery of a therapeutic for up to two weeks.

## Introduction

Treatment for site-specific diseases, such as cancer^1^, atherosclerosis^2^, and restenosis of blood vessels^3^, suffer from the inability to control and target the delivery of potent therapeutics due to the reliance on systemic drug delivery. To overcome this challenge, novel approaches in drug encapsulation^4^ and molecular therapies^5^ have been extensively explored as a means to reduce off-target side effects by providing local drug delivery. Recently, ‘smart’ drug delivery systems that respond to an internal or external stimulus (e.g., redox^6^, pH^7^, temperature^8^, magnetic^9^, light^10^, and sound^11^) have been proposed as a means to provide targeted release of a therapeutic. Though these stimuli-responsive systems have demostrated enhanced delivery efficiency and efficacy, their potential remains limited due to the reliance on diffusion of drug from the blood vessel into the diseased tissue. As a result, there is limited distribution of the therapeutic within the diseased tissue, a challenge commonly found in the treatment of the aforementioned site-specific diseases. Therefore, there is a need for a means to not only provide targeted drug release, but to also improve the penetration and distribution of the drug to maximize therapeutic benefits.

For decades, ultrasound-enhanced drug delivery has been reported to provide targeted drug delivery with improved therapeutic outcomes for diseases that face physical barriers to drug transport such as the blood brain barrier^12^, skin^13^, and heightened resistance to diffusion in cancers^14^. Ultrasound is a non-invasive and cost effective diagnostic and therapeutic modality that uses high-frequency sound waves to induce thermal and mechanical effects^15^. It is already under use as a means to non-invasively destroy cancerous tissue^16^, promote drug delivery by releasing payloads from various carriers^17^, and temporarily permeabilize the endothelium to allow the passage of small molecules^12,18^. For drug delivery, cavitation agents are typically used to nucleate cavitation and enable mechanical forces (microstreams, shear forces, jetting, and shockwaves) that enhance drug uptake across cells and tissue^19,20^. Cavitation nuclei such as microbubbles^21^, phase-change nanodroplets^22,23^, and non-biodegradable polymer nanocups^24^ localize drug release and improve drug penetration and distribution for therapeutics co-administered or directly loaded within the particle. Recently, solid cavitation agents have garnered increased interest owing to their ability to sustain cavitation for minutes and able to penetrate into tissue^25^. However at present, solid cavitation agents are non-degradable and thus provide limited capacity for drug release. It is therefore desirable to combine the targeted drug release of microbubbles with the sustained cavitation provided by solid cavitation nuclei in order to address the specific needs for addressing difficult to treat site-specific diseases. Yet, there are no degradable polymer-based drug delivery vehicles capable of nucleating cavitation from surface stabilized bubbles, despite the established research in polymer drug-delivery systems. The key challenge here is the need for surface cavities; current polymer drug-delivery vehicles are predominantly smooth spheres. Therefore, we propose here that these needs are met with an “all-in-one” drug-loadable biodegradable solid cavitation nuclei that sustains cavitation across minutes, remain at the diseased site after exposure to ultrasound, and locally release a payload across several days.

In our report, we modified a double emulsion method and lyophilization procedure to manufacture novel uniform multi-cavity poly-co-lactic-co-glycolic acid microparticles (mcPLGA MPs) with trapped gas pockets. Both rhodamine B (RhB) and 4′,6-diamidino-2-phenylindole (DAPI) were loaded into the mcPLGA MPs to represent a small molecule therapeutic agent and to track release rates from the RhB-mcPLGA MPs. Release rates were investigated within solution and after implantation into a tissue mimicking model using high intensity focused ultrasound (HIFU). RhB-mcPLGA MPs were remotely implanted into the sub-endothelial space of *ex vivo* porcine arteries without observable damage to the endothelium and underlying smooth muscle cells during exposure to HIFU. Once implanted, the RhB/DAPI-mcPLGA MPs hydrolytically degraded and released both RhB and DAPI. Though free RhB was not detectable, DAPI was observed to stain the nucleus of cells after one day, reaching far from the location of the implanted particles after 3 days.

## Results and Discussion

### Formation and physical characterization of RhB-mcPLGA MPs

**Figure 1:**
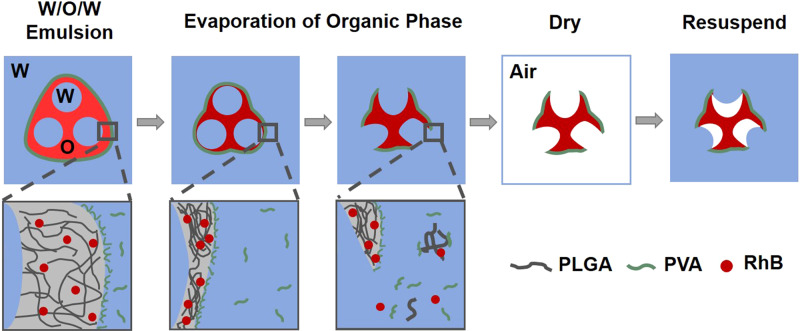
Formation of RhB-mcPLGA MPs using a modified water-organic-water (W/O/W) double emulsion method. Here, multiple internal sub-micron aqueous droplets are formed within a single organic droplet comprised of dichloromethane (DCM), PLGA, and RhB. The surrounding water phase is comprised of polyvinyl alcohol (PVA), which self assembles onto the O/W interface and stabilizes the multi-core droplet. During the evaporation of the DCM, thin edges of PLGA are formed at the apexes of the multi-core droplet. These thin edges eventually are ruptured, releasing the aqueous cores into the surrounding medium and forming the multi-cavity structure.

Figure 1 illustrates our hypothesized approach for manufacturing RhB-mcPLGA MPs. Here, we used a modified water-organic-water (W/O/W) double emulsion method that traps multiple aqueous droplets into the organic phase, thereby forming a multi-core aspherical water-organic (W/O) droplet^26^. The W/O droplet comprised of an organic phase of dichloromethane (DCM) with dissolved PLGA and RhB encapsulating deionized water droplets. PLGA was selected as the polymer because it is well established that this polymer is biocompatible, biodegradable, and capable of drug loading across a broad range of therapeutics^27^. Furthermore, PLGA has already been FDA approved as a drug delivery vehicle for numerous diseases^28^. Here, RhB was used to represent small molecule therapeutic agents, track the location of the particles, and measure payload release rates. The W/O droplets were surrounded by a continuous phase comprised of an aqueous solution of polyvinyl alcohol (PVA), which self-assembled onto the interface and stabilized the droplet^29^. During heating, the DCM evaporated from the solution, causing the thin edges at the apexes of the aspherical droplet to rupture. After the DCM was completely removed, a multi-cavity PLGA structure remained. In order to trap gas within these cavities, the aqueous phase was removed by freeze drying. After suspending the dried RhB-mcPLGA MPs into an aqueous medium, nanobubbles were trapped within the cavities^24,30^.

**Figure 2:**
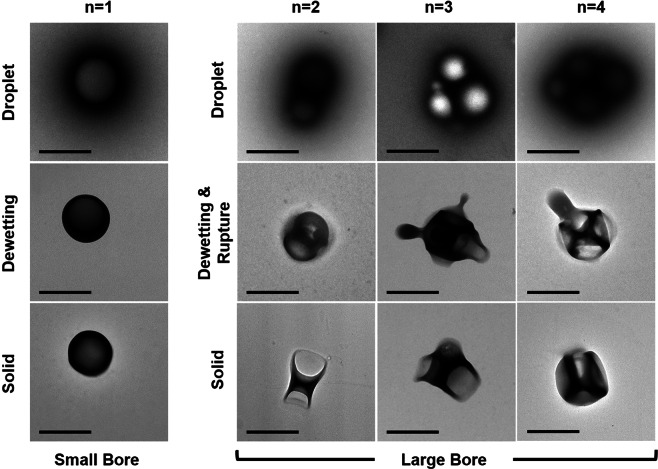
Representative TEM images showing the formation of RhB-hsPLGA and RhB-mcPLGA MPs. The scale bars represent 1 μm.

Figure 2 shows transmission electron microscopy (TEM) images of different stages of development for double emulsion droplets with different number of aqueous cores. In order to control the number of aqueous cores within the organic phase, we used two homogenizers with different dispersing bore diameter. A homogenizer (IKA T25) with a small bore diameter (8 mm) created W/O/W droplets with a single aqueous core. These spherical droplets displayed no asymmetries in the structure. No cavities were formed from these droplets. Instead a hollow spherical RhB-loaded PLGA microparticle (RhB-hsPLGA MP) was formed, which was further validated with scanning electron microscopy (SEM) and fluorescent imaging (Fig. S1). The RhB-hsPLGA MPs were uniform in diameter before (1.08 ± 0.17 μm) and after drying and suspension (1.11 ± 0.12 μm) into phosphate buffer saline (PBS).

Using a Silverson L5M-A homogenizer with a larger bore diameter (16 mm), W/O/W emulsions with two or more aqueous cores were generated (Fig. 2). These droplets subsequently deformed to create polyhedral microparticles with multiple surface cavities. Approximately two to five submicron water droplets were observed to be surrounded by an aspherical PLGA-laden solution of DCM. The PLGA formed a shell around the droplet as the DCM evaporated, which caused the DCM to dewet from the interface. During this process, the thinner surfaces at the apexes of the aspherical droplet ruptured, releasing the water droplets into the continuous phase. After rupture, the remaining DCM evaporated off, leaving behind a PLGA multi-cavity structure.

**Figure 3:**
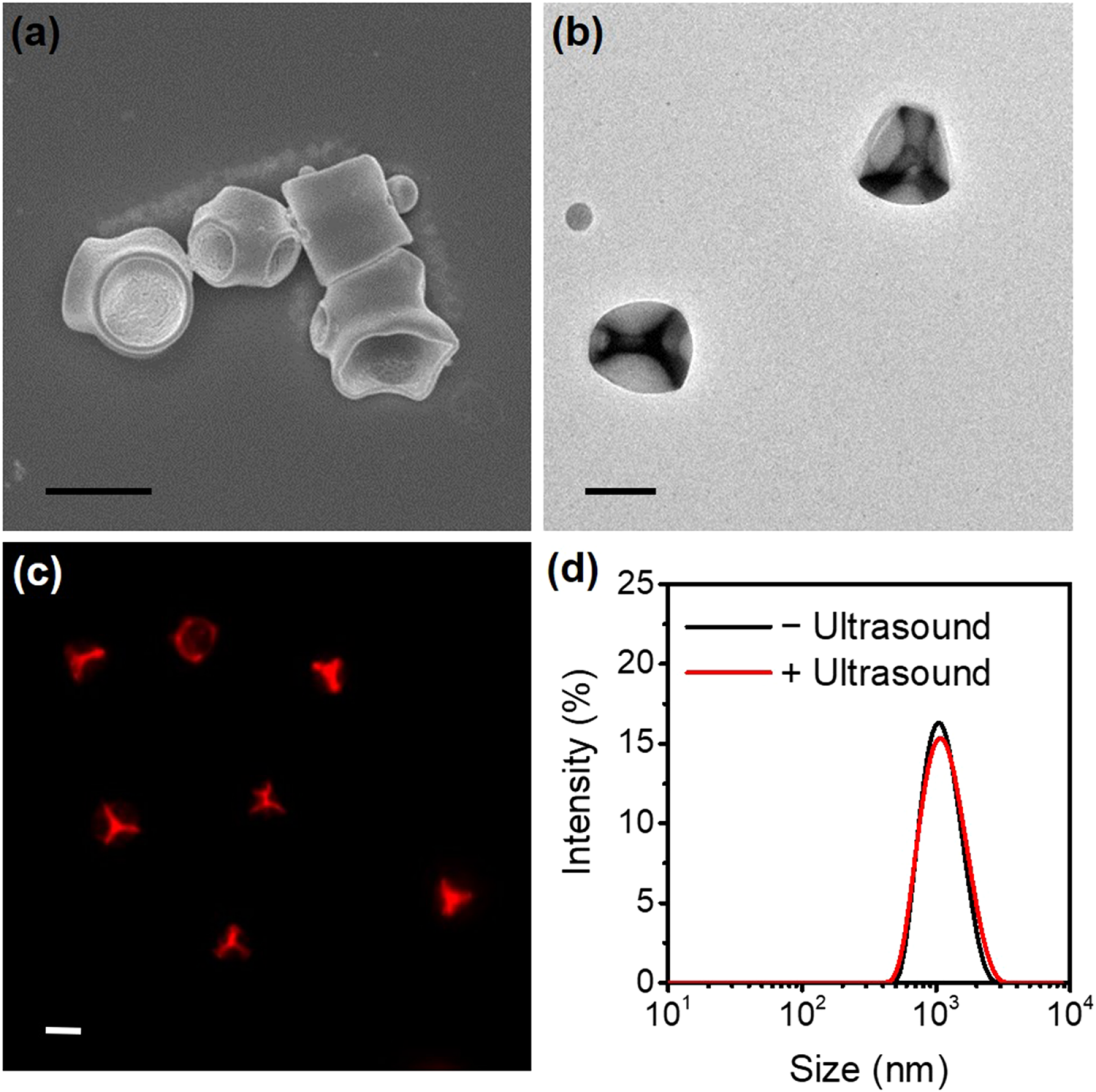
(**a**) Representative SEM, (**b**) TEM, and (**c**) fluorescence images of RhB-mcPLGA MPs. (**d**) Size distributions of RhB-mcPLGA MPs before and after exposure to HIFU. The scale bars represent 1 μm in (**a**–**c**).

Representative SEM and TEM images of RhB-mcPLGA MPs (Fig. 3) emphasized the uniformity of the polyhedral structure. Three to four cavities (with a mean size of 0.71 ± 0.25 μm) per RhB-mcPLGA MPs were observed to be the most common. Side and bottom views of RhB-mcPLGA MPs (Fig. S2) present a smooth particle surface with rough cavity inner surface area, which may contribute to gas trapping. Figure 3c shows fluorescent images of RhB-mcPLGA MPs, indicating the successful loading of RhB into the PLGA components of the microparticle with an encapsulation efficiency of 61.2 ± 7.9% as measured by the UV-absorbance of RhB at 553 nm. Considering that PLGA has already been established as an ideal polymer for high-capacity drug loading into PLGA-based spherical particles^31–33^, our particles will also be capable of drug loading (as further evidenced by the presence of RhB). The size distribution of RhB-mcPLGA MPs did not change before or after lyophilization and remained at a hydrodynamic diameter of 1.15 ± 0.32 μm. Furthermore, there was no appreciable change in size after exposure to HIFU (Fig. 3d). No agglomerations or aggregates were observed after suspension of the dry powder in deionized water or PBS.

### Degradation of RhB-mcPLGA MPs and release of payload

**Figure 4:**
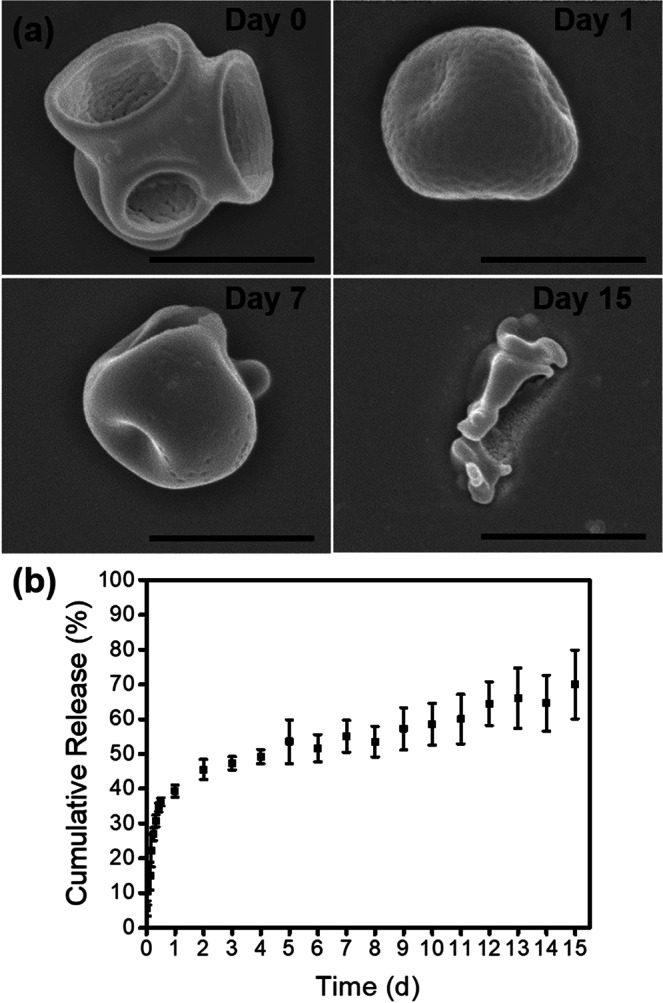
(**a**) SEM image of RhB-mcPLGAs incubated in PBS at 37 °C across 15 days. The scale bars represent 1 μm. (**b**) Release profile of model drug (RhB) from RhB-mcPLGAs MPs. The bars represent the standard deviation for 5 independent samples.

We next measured the degradation and release kinetics of the RhB-mcPLGA MPs in PBS at 37 °C with or without exposure to HIFU. SEM images (Fig. 4a) of mcPLGA MPs suspended in a PBS solution for 15 days revealed a rapid degradation of the edges of the cavities, resulting in morphological changes of the particles within one day, though changes in the diameter of the particles were not appreciable until after seven days (Fig. S3). At day 15, the particles began to aggregate in solution as indicated by an increase in particle diameter with a broad size distribution.

The release of RhB from RhB-mcPLGA MPs in PBS at 37 °C across 15 days is shown in Fig. 4b. We observed an initial burst release of 34.7 ± 1.2% of encapsulated RhB within the first 24 hours. After 24 hours, there was slow and sustained release of RhB to 70.0 ± 1.0% at day 15. This second-order release rate was in stark contrast to the first-order release rates typically found with spherical PLGA particles^34^. Therefore, it is plausible that the rapid degradation of the edges of the cavity with a larger surface area to volume ratio contributed to the observed burst release of RhB within the first 24 hours. Interestingly, exposure to HIFU did not facilitate the release of RhB (Fig. S4), which corresponded the lack of appreciable degradation of the mcPLGA particles (Fig. 3d). This confirmed that release of RhB was predominantly due to the hydrolytic degradation of the PLGA.

### Acoustic characterization of RhB-mcPLGA MPs

**Figure 5:**
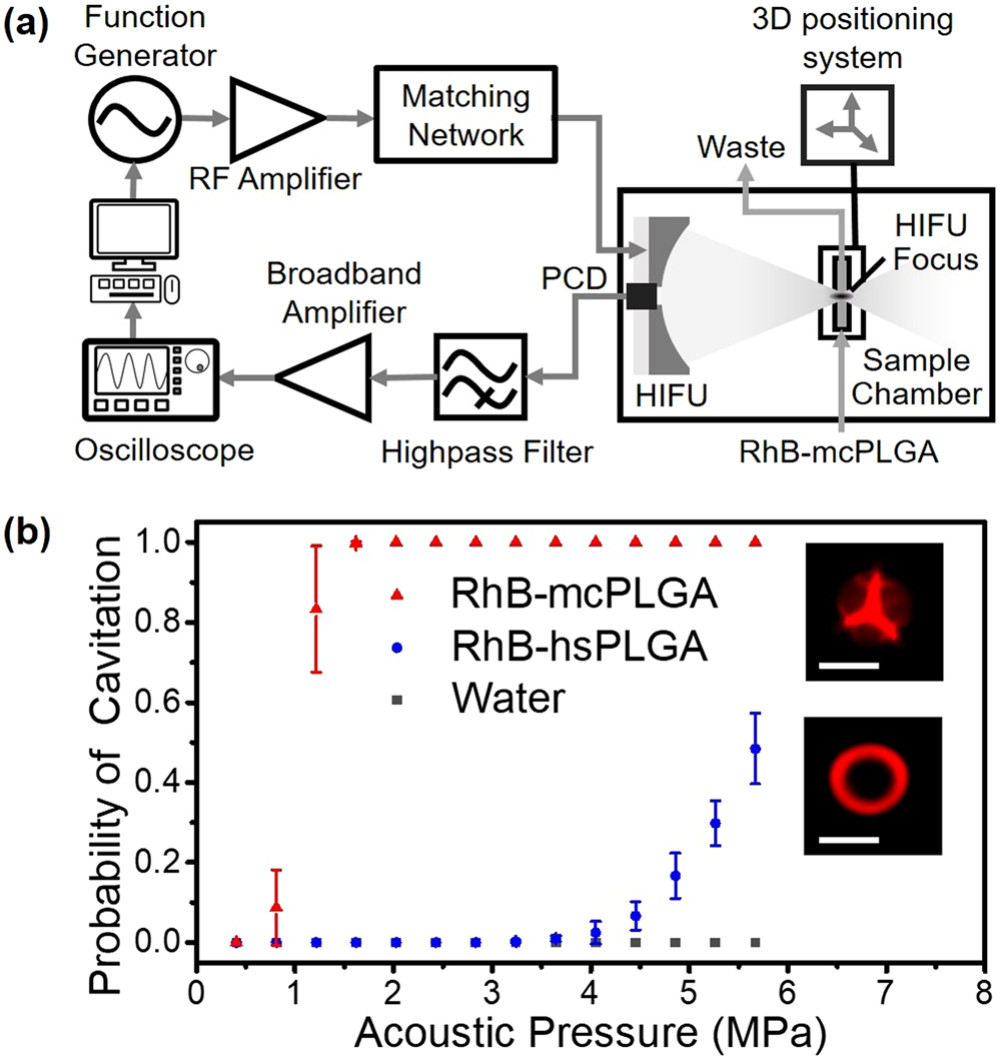
(**a**) Schematic of the HIFU setup. (**b**) Probability of cavitation of water, spherical PLGA particles, and RhB-mcPLGA MPs exposed to HIFU at 1.1 MHz. (**Inset**) Fluorescence images of representative RhB-mcPLGA MPs and RhB-hsPLGA MPs. The scale bars represent 1 μm. The points are an average and standard deviation of 3 independent samples.

The acoustic responses of RhB-mcPLGA MPs were evaluated using a conventional HIFU transducer setup in a degassed water tank (Fig. 5a). Dried RhB-mcPLGA MPs were suspended in degassed water at various concentrations ranging from 0.01 mg/ml to 1 mg/ml and pumped through an agarose flow phantom with a 1.6 mm diameter channel at a rate of 0.2 ml/min (Fig. S5).

During exposure to HIFU across a range of pressure amplitudes, broadband emissions from RhB-mcPLGA MPs were detected with a passive cavitation detector (PCD). From the power spectral density curves (Fig. S6) we detected a broadband dominant signal, suggesting the presence of inertial cavitation at all pressure amplitudes tested that provided a signal. The likelihood for an inertial cavitation event to occur was measured for increasing pressure amplitudes, and the inertial cavitation threshold (i.e., a probability for an event to occur equals 50%)^35^ was determined to be 1.1 MPa peak negative pressure amplitude (Fig. 5b) for RhB-mcPLGA MPs at a concentration of 1 mg/ml. Inertial cavitation thresholds increased from 1.1 MPa peak negative pressure (at 1 mg/ml) to 1.8 MPa and 2.4 MPa peak negative pressures when diluted by 10 fold and 100 fold respectively (Fig. S7), suggesting that the likelihood for an inertial cavitation event to occur was proportional to the number of gas nuclei within the acoustic focus. For a given dose of particles, RhB-mcPLGA MPs provided inertial cavitation at similar pressure amplitudes to previously reported single-cavity non-degradable polymeric particles under comparable HIFU exposure conditions^24^. Without flow, RhB-mcPLGA MPs sustained cavitation for up to 10 min (1.9 MPa, 5% duty cycle, 2 Hz pulse repetition frequency; Fig. S8) similar to smaller non-degradable polymeric particles, enabling more time for a therapeutic to be delivered into the given target^25^.

It is crucial to note that RhB-hsPLGA MPs with the same size (Fig. 5b (inset)) and concentration did not exhibit measurable inertial cavitation until 3.5 MPa peak negative pressure with a cavitation threshold approaching 5.5 MPa peak negative pressure. Therefore, we concluded that that the presence of nanobubbles within the cavities were crucial for the nucleation of cavitation, which was consistent with previous observations^24,36,37^.

### Implantation of RhB-mcPLGA MPs and release of RhB in an agarose flow phantom

**Figure 6:**
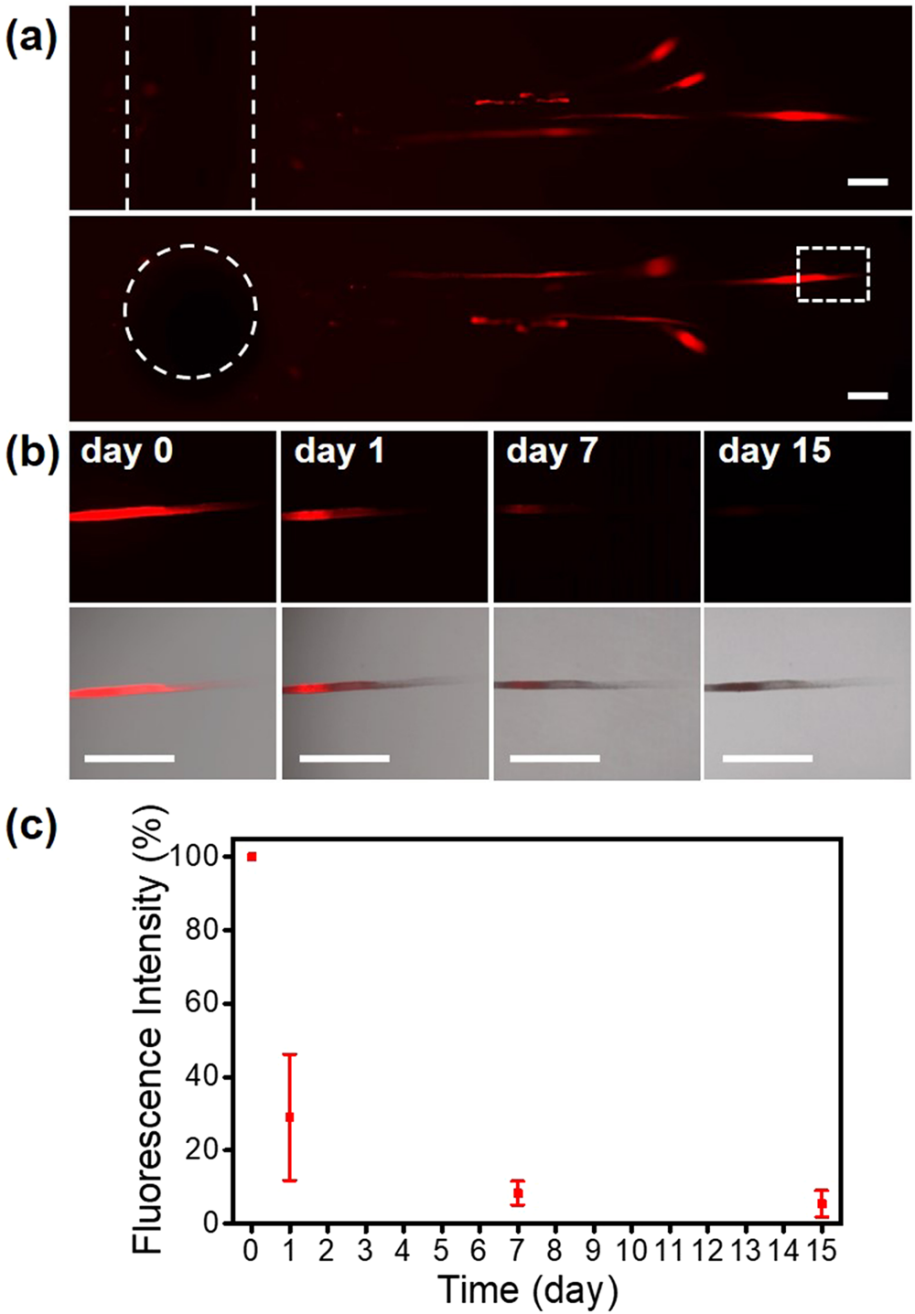
Microscopy images of RhB-mcPLGAs embedded in tissue-mimicking agarose phantoms. (**a**) Representative fluorescent images of the phantoms sliced horizontally and radially to the channel (1.6 mm). (**b**) High resolution of fluorescence, and merged images across 15 days. (**c**) Quantitative analysis of change in fluorescence intensity across 15 days. The points are an average and standard deviation of 3 independent samples. The scale bars represent 500 μm in (**a**,**b**).

After determining the inertial cavitation threshold for RhB-mcPLGA MPs, the ultrasound enhanced delivery of these particles was evaluated in an agarose flow phantom. Agarose was selected as the phantom material because the acoustic impedance mismatch between agarose and water was negligible, effectively making agarose acoustically transparent in water and not allowing for the formation of standing waves in our setup^38^. Figure 6a shows fluorescence images of a representative agarose flow phantom both horizontally and radially after 10 min of ultrasound exposure. RhB-mcPLGA MPs penetrated beyond the lumen of the channel with an average distance of 4.29 ± 1.19 mm. The accumulation of RhB-mcPLGA MPs always occurred in the direction away from the HIFU transducer. As the RhB-mcPLGA MPs propelled into the agarose phantom, large tunnels (20.4 ± 2.5 μm in diameter) were formed. Previous work has shown that bubbles nucleated from surface cavities by HIFU at frequencies of 1.6 MHz, have a peak diameter of approximately 20 μm in diameter^39^. Therefore, we hypothesize that these tunnels were formed from burrowing cavitation bubbles, and the size of the tunnel was approximately the maximum size of the bubbles formed from the RhB-mcPLGA MPs^40^. We also noted that these tunnels were only observed in the agarose flow phantom model, which was similar to other studies^41^. Such tunnels were not present in the porcine artery (which will be further discussed in Section 2.6), nor have they been observed in other animal tissue models^24^. Therefore, these tunnels are unique to gel-based models, possibly due to the homogenous structure of the polymeric matrix, and suggest that delivery into tissue undergoes a different mechanism.

Our results suggest that RhB-mcPLGA MPs are drug carriers capable of ultrasound-mediated implantation into agarose phantoms. We thus measured the *in situ* release of RhB from RhB-mcPLGA MPs embedded in agarose flow phantom by incubating the sectioned agarose flow phantom in PBS at 37 °C (Fig. 6b) across 15 days. The fluorescence intensity was quantified and compared to the initial intensity as shown in Fig. 6c, and was also observed to be proportional to the exposure time of the camera (Fig. S9). After 24 h incubation, the fluorescence intensity exhibited a sudden decrease of fluorescence, which was in agreement with our earlier observations on RhB release trend, i.e., a burst release of RhB after one day followed by sustained released across 15 days (Fig. 4b).

Here, we attributed the decrease in fluorescence intensity to the release of RhB from the RhB-mcPLGA MPs. As the PLGA degrades, released RhB diffused rapidly through the agarose gel^42,43^, and resulted in a substantial dilution of the free RhB with an undetectable fluorescence compared to the RhB trapped in the particles. It is unlikely that this decrease in signal was due to photobleaching because RhB was photostable for several hours of illumination time^44^, which far exceeded the illumination time exposed to the samples. Furthermore, an independent set of samples were incubated at 4 °C, and displayed a drastically slower decay rate (Fig. S10); the degradation of PLGA was proportional to temperature^45^. This result further confirmed that the decay in signal was primarily due to the loss of PLGA and not from photobleaching.

### Sub-Endothelial implantation of RhB/DAPI-mcPLGA MPs in porcine artery

**Figure 7:**
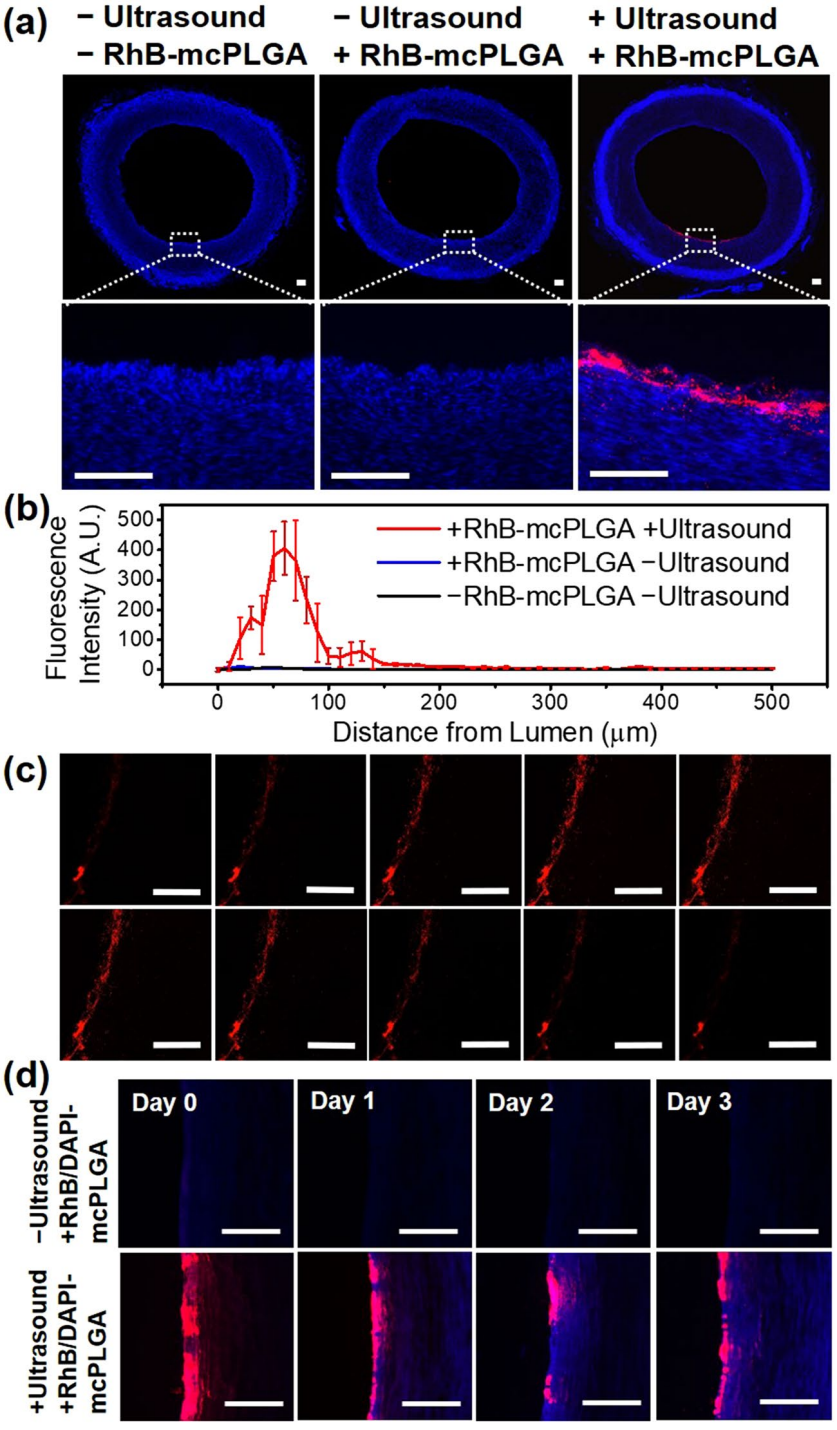
(**a**) Fluorescent images of DAPI stained porcine arteries under different treatment conditions. RhB-mcPLGA MPs (red) are found to be implanted into the sub-endothelial spaces only after exposure to HIFU. The dotted box indicates the location of the zoomed in image below. (**b**) Fluorescence intensity profile of RhB-mcPLGA MPs across the thickness of the artery. Data shown is the mean fluorescence intensity of triplicate arteries. The points are an average and standard deviation of 3 independent samples. (**c**) CLSM images of different longitudinal sections of a representative porcine artery treatment. (**d**) Representative fluorescence images of porcine arteries showing the distribution of RhB/DAPI-mcPLGA MPs and DAPI released from mcPLGA MPs from day 0 to day 3. The scale bars represent 200 μm. RhB-msPLGA MPs were indicated by red and DAPI fluorescence by blue.

We sought to demonstrate site-specific delivery of mcPLGA MPs to the sub-endothelial space of a blood vessel where arterial disorders are typically found. Considering that the HIFU beam focal volume was too large to fit within the arteries of a mouse and that the arteries of a mouse are orders of magnitude smaller than that of a human, we deemed it impractical to use a murine model. Instead, *ex vivo* porcine arteries were selected as the arterial model owing to their relative size and structure more closely resembling that of a human artery compared to the agarose phantom. The lumen of the *ex vivo* porcine arteries were perfused with either RhB-mcPLGA MPs or RhB/DAPI-mcPLGA MPs and exposed to HIFU. Fluorescent images of porcine arteries (Fig. 7a) post treatment indicated that RhB-mcPLGA MPs were embedded into the artery only after HIFU exposure without the presence of substantial heating (Fig. S11). Figure 7b shows the fluorescence intensity profile as a function of depth from the endothelium, with a maximum accumulation of RhB-mcPLGA MPs at a depth of 60 μm from the endothelium. This result clearly indicated that RhB-mcPLGA MPs were implanted primarily into the sub-endothelial space of *ex vivo* porcine arteries avoiding possible damage of the underlying smooth muscle cells during exposure to HIFU.

Similar to the agarose flow phantom, the accumulation of RhB-mcPLGA MPs occurred in the direction away from the HIFU transducer, confirming that HIFU was able to target specific regions of the artery. Furthermore, the chord length of the region embedded with RhB-mcPLGA MPs (1.41 ± 0.28 mm) was the same length as the full width half maximum of the HIFU focus (1.37 mm), indicating that the delivery of particles was restricted to the focus of the HIFU (Fig. S12).

In order to ensure that our results were not due to experimental contamination during sectioning of the porcine artery, confocal laser scanning microscope (CLSM) images across the longitudinal depths of the artery were obtained. These Z-stack images (Fig. 7c) validated that all of RhB-mcPLGA MPs were in fact implanted. Thus, the penetration depth of RhB-mcPLGA MPs was not a result of the sectioning methods used, but instead solely from acoustic cavitation-mediated transport.

To directly show the ability to release a potential therapeutic agent, porcine arteries were perfused with RhB/DAPI-mcPLGA MPs and were exposed to HIFU at day 0. The arteries were then flushed with water and allowed to rest for up to three days. Fluorescent images (Fig. 7d) of control arteries (i.e., not exposed to HIFU) showed no presence of fluorescent particles and no presence of DAPI staining, suggesting that there was no delivery present. In stark contrast, there was an abundance of RhB/DAPI-mcPLGA MPs in the subendothelial space, similar to Fig. 7b, after exposure to HIFU. At day 0, DAPI was not observable because DAPI must bind to DNA in order to provide detectable fluorescence. After one day, DAPI was observed in the cells neighboring the implanted RhB/DAPI-mcPLGA MPs. At days 2 and 3, there was a strong DAPI signal deep throughout the arterial wall. It was also evident that the depth of DAPI penetration increased with each day. Here, the only source of DAPI was from the HIFU-implanted RhB/DAPI-mcPLGA MPs. Once released, DAPI diffused through the arterial wall, binding to DNA in the nucleus. Considering that DAPI is only fluorescent at detectable levels after it binds to DNA, the presence of DAPI far from the lumen of the artery indicated that DAPI must have been released from the particles, showing the drug delivery capabilities of these particles long after HIFU-implantation.

Here, we showed the ability to actively target the remote implantation of a drug delivery vehicle into arterial tissue. Though this study was limited to an *ex vivo* system and therefore cannot account for the physiological environments present in an *in vivo* model, our current results marked a step-change from conventional passive delivery strategies reliant on diffusive mechanisms and other ultrasound-enhanced drug delivery strategies reliant on short-term drug delivery. Furthermore, the ultrasound transducers used in these studies may be translated to larger *in vivo* animal models; similar HIFU transducers have already been applied to non-human primates and pigs for various therapeutic applications^46^. Passive targeting strategies to accumulate drug loaded nanoparticles are still unable to retain the particles entirely inside the diseased area and may lead to non-specific accumulation in undesired location due to long circulation times. With regards to ultrasound-enhanced drug delivery, microbubbles or other cavitation nuclei are often decoupled from the drug delivery vehicle, limiting the potential for increased distribution and depth of penetration of the therapeutic^24^. Furthermore, studies have shown that solid cavitation nuclei travel further than the co-administered therapeutic agent in different tissue models^24,47^. Here, we have coupled the therapeutic with the solid cavitation nuclei, opening up the potential for precise targeting of drug delivery by modulating HIFU parameters remotely. As a drug delivery vehicle actively driven by focused ultrasound, we are able to remotely implant these devices into arterial tissue and deliver a model therapeutic far from the location of the implantation. Other tissue such as the skin or dense tumors may also be targets in the future.

### Histopathological analysis by H and E staining

**Figure 8:**
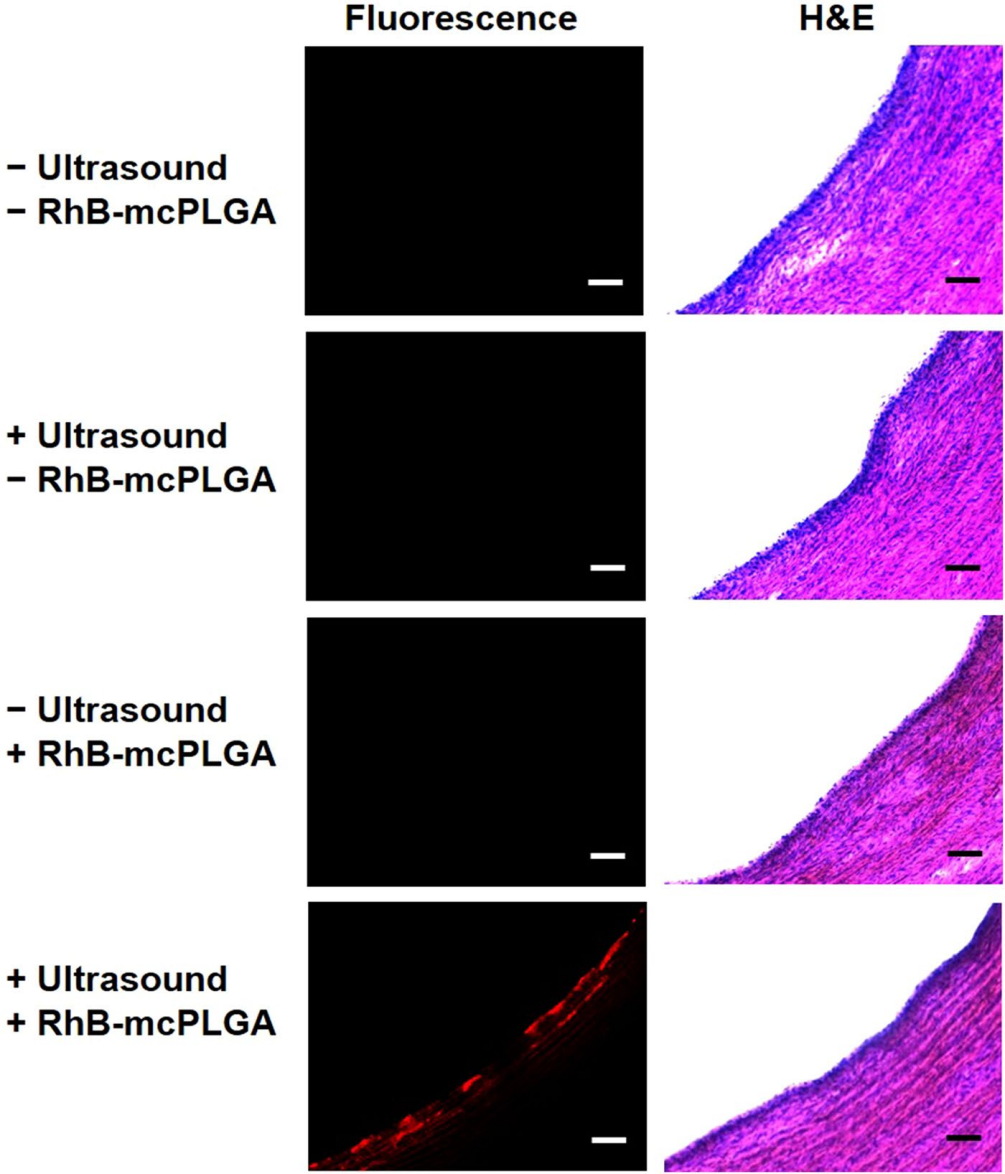
Histopathological analysis by H&E staining of porcine arteries under different treatment conditions. The scale bars represent 100 μm.

From our observations of tunnel formations in the agarose gel, we considered the potential for HIFU-guided implantation of RhB-mcPLGA MPs to destroy or rupture the integrity of the blood vessel. We therefore conducted histopathological analysis of the RhB-mcPLGA MPs at the site of implantation to observe the effects of mechanical stress induced by HIFU or inertial cavitation. From Fig. 8, it was evident that implantation of RhB-mcPLGA MPs into the porcine artery occurred without any morphological damage to the endothelial region. Note, however, that these histopathological images cannot distinguish between potentially released RhB and RhB still bound within the mcPLGA MPs. Endothelial cells (eosin stained inner blue region) showed no signs of coagulative necrosis and interestingly no observable tearing of tissue lying underneath in comparison to control groups.

It is plausible that cavitation provided substantial shear stress on the endothelium^12,18,24^ to temporarily open gaps between endothelial cells and allow for extracellular convective transport of the RhB-mcPLGA MPs. Furthermore, the RhB-mcPLGA MPs were not randomly distributed in the sub-endothelial space but were instead primarily accumulated within the spaces between the lamella of the cells at the wall away from the HIFU transducer. This observation may be attributed to the cavitation from the particles during HIFU exposure. These cavitation events likely created streaming effects near the endothelium, and in combination with acoustic radiation forces from the HIFU, particles will have been pushed away from the HIFU transducer. This phenomenon was also observed by others using different solid cavitation agents^48^. Without the presence of any noticeable mechanical or cellular damage, HIFU-mediated implantation of RhB-mcPLGA MPs just beyond the tunica intima was deemed safe and without any immediately presentable adverse side-effects.

## Conclusion

Here, we report on the fabrication of a drug-loadable biodegradable multi-cavity cavitation nuclei (RhB-mcPLGA MPs) for HIFU-mediated implantation into tissue. The RhB-mcPLGA MPs were synthesized using a modified double emulsion method, which provided multiple internal aqueous cores within an aspherical organic droplet containing the payload and PLGA. These aspherical droplets formed into the multi-cavity particles with a uniform size and shape with rough hydrophobic cavities. After gas trapping, RhB-mcPLGA MPs nucleated cavitation under exposure to HIFU at clinically relevant exposure conditions and was able to sustain cavitation for up to 10 minutes. RhB-mcPLGA MPs were remotely implanted with HIFU into an agarose gel at depths of up to 7 mm. Once implanted, sustained release of RhB across 15 days was observed. These particles were also implanted with HIFU into the sub-endothelial space of porcine arteries without damaging either the intima or media at the site of implantation. The delivery of the particles matched the beam width of the acoustic focus and was observed to be unidirectional, suggesting that this technique may be tuned to the ultrasound beam form. In summary, we have developed a platform technique for the remote implantation of loadable biodegradable particles capable of sustaining drug delivery at the site of interest. This novel and innovative method opens up a non-invasive route to improve treatment for site-specific diseases as a result of tumor, peripheral arterial disease, wound infections, or other diseases that require transdermal administrations.

## Experimental Section

### Materials and reagents

Poly(lactide-co-glycolide 50:50) (PLGA, ResomerRG504 H), Poly(vinyl alcohol) (PVA) (Mw 9,000–10,000, 80% hydrolyzed), Dichloromethane (anhydrous, > = 99.8%), Rhodamine B, Phosphate buffered saline (PBS), and Fluoroshield™ with DAPI were purchased from Sigma-Aldrich and used as received. Agarose was bought from Vivantis Technologies. Deionized water was obtained from a pure water system (Stakpure, Germany). Pig arteries were obtained from an abattoir (Primary Industries, 2 Buroh lane, Singapore). Tissue-Tek O.C.T. compound was purchased from Sakura Finetek (Torrance, USA). All sample chambers and HIFU transducer holders were built in house.

### Preparation of RhB-hsPLGA, RhB-mcPLGA, and RhB/DAPI-mcPLGA MPs

RhB loaded hollow sphere and multi-cavity PLGA microparticles (RhB-hsPLGA and RhB-mcPLGA MPs respectively) were prepared by a modified water/organic/water double emulsion solvent evaporation process^26^. 50 mg of PLGA and 0.5 mg of RhB was dissolved in 2 mL of dichloromethane (DCM). Then 100 μl of deionized water was added to the PLGA solution and sonicated (Ultrasonic processor VCX 130, Sonics and Materials Inc., USA) at 100 W for 30 s in an ice bath to form an emulsion. The obtained W/O emulsion was then poured into a 5% PVA solution and homogenized over ice for 5 min. To form the RhB-hsPLGA MPs, an Ultra-turrax (IKA T25, Germany) with an 8 mm dispersion tool (Model S10N-8G) was used. To form the RhB-mcPLGA MPs, we applied a Silverson Lab mixer (L5M-A, Silverson, MA), with a 5/8” MICRO tubular mixing unit. The particle suspension was then stirred at 40 °C for 1 h in a chemical fume hood to allow organic solvent evaporation. The PLGA particles were collected by centrifugation at 1,000 G for 5 min, and subjected to three cycles of centrifugation, wash, and dispersion in water. Fresh microparticles were frozen at −80 °C, and then lyophilized using a lyophilizer (Alpha 2–4 LSCbasic, Christ, Germany) for 48 h. After drying, particles were suspended in PBS immediately prior to use. For fabrication of RhB/DAPI-mcPLGA MPs, 0.5 mg of DAPI was added before sonication and homogenization following the method described above.

### Acoustic setup

A 1.1 MHz high intensity focused ultrasound (HIFU) transducer (Sonic Concepts H107) was used for acoustic excitation. A 7.5 MHz passive cavitation detector (PCD) (V320, Olympus, Singapore), co-axially and co-focally aligned with the HIFU transducer, was used for detection of acoustic emissions at the HIFU focus. The HIFU transducer was calibrated using a 0.2 mm needle hydrophone (Precision Acoustics SN2562). The geometric focus of the transducer was 1.37 mm in width and 10.21 mm in length. The HIFU transducer was driven by a function generator (Keysight 33210 A) and a RF power amplifier (Electronics & Innovation 1040 L). All experiments with HIFU were carried out in a large tank filled with filtered, degassed and deionized water. Acoustic amplitudes in this study are reported in MPa peak negative pressure amplitudes.

### Characterization of microparticles

Size and surface morphology of RhB-hsPLGA and RhB-mcPLGA MPs were assessed using a JEOL JSM-6700 Field Emission Scanning Electron Microscope (FE-SEM; JEOL Ltd.) at an acceleration voltage of 5 kV. Samples for the SEM were prepared by dropping 10 μl of 1 mg/ml suspension on silica wafers and air drying. For observations, the wafers were mounted onto a metal stub using double-sided electrical tape, and coated with platinum (JEOL, JFC 1600 Auto Fine Coater, Japan) for 3 min at 20 mA. All images were recorded under Secondary Electron Imaging (SEI) mode. Transmission electron microscopy (TEM) was performed using a JEOL JEM 1400 operating at 120 kV. Samples for TEM imaging were prepared by adding 10 μl of aqueous dispersions on a 300-mesh carbon coated copper grids. The grids were air dried at room temperature. Size distributions were determined by dynamic light scattering (DLS) (Malvern Nano-ZS). To determine size changed due to HIFU, samples were exposed to 10 min of 1.1 MHz HIFU at 10% duty cycle and 3.9 MPa peak negative pressure amplitude. Fluorescence images of RhB-mcPLGA MPs were collected using a Zeiss AxioVert 200 Inverted Fluorescence Microscopy.

The quantity of RhB present was calculated according to the UV-absorbance of RhB at 553 nm measured by a UV-Vis Spectrometer (Shimadzu UV 2450). A standard curve with a concentration range of 1 to 10 μM was made in PBS to correlate the mass of RhB in solution with the UV-absorbance spectral curve. The encapsulation efficiency was calculated by first measuring the remaining RhB within in the supernatant of RhB-mcPLGA MPs after solvent evaporation and subtracting it from the total amount of RhB added into the system. This difference was divided by the total amount of RhB added and multiplied by 100 to obtain the percent of RhB encapsulated.

### Acoustic characterization of microparticles

An acoustically transparent agarose sample chamber was first made from a 1% (w/v) of agarose solution, which was boiled and degassed for 30 min to prevent cavitation as a result of endogenous bubbles. The agarose solution was then poured into a bespoke cuboid mold (50 mm in length × 30 mm in width) and sealed with acoustically transparent mylar windows. A 1.6 mm steel rod was threaded through the mold. After gelation was completed, the rod was removed, creating a flow channel. The agarose phantom was submerged in the degassed water tank and aligned to the focus of the transducers. With the channel filled with air, the PCD was driven with a pulser-receiver (JSR Ultrasonics DPR300) to determine the position of the channel. A 3D positioning system was used to adjust the chamber until the channel was at the focus of the HIFU transducer. A 1 mg/ml suspension of RhB-mcPLGA MPs were flowed through the channel using a syringe pump at a rate of 0.2 ml/min for ultrasound exposures. The HIFU transducer was driven by a function generator (Keysight 33210 A) and a RF power amplifier (Electronics & Innovation 1040 L). RhB-mcPLGA MPs were exposed to 20 cycle bursts with increasing peak negative pressure amplitude at a pulse repetition time of 0.5 s. Acoustic emissions from mcPLGA MPs were detected using a 7.5 MHz PCD co-axially aligned with the HIFU transducer. The PCD output was passed through a 2.5 MHz high-pass filter and amplified using a broadband preamplifier (Stanford Research Systems SR445A). The received signals were then recorded onto an oscilloscope (Keysight DXOX3032A) and post processed to determine the power spectral density (PSD) curve. For each burst, the area under the PSD curve was determined and compared to degassed water exposed to HIFU under the same conditions. Following the signal processing, cavitation was said to occur if the received signals were 10 dB higher than noise from the water control^24,25^. The probability of cavitation was determined as the percentage of bursts that recorded a cavitation event out of the total number of HIFU bursts (120 bursts).

### Study of degradation of mcPLGA MPs and release of RhB

RhB-mcPLGA MPs were suspended in PBS (pH 7.4) and incubated at 37 °C with stirring. Particle size distribution was measured at predetermined time points using DLS. Size and morphology of RhB-mcPLGA MPs were further characterized using FE-SEM as described earlier.

The release of RhB in solution was performed using a sample and separation method^49^. 50 mg of freeze-dried RhB-mcPLGA MPs was collected and dispersed in 50 ml of 0.01 M PBS (pH 7.4) buffer solution in sealed vials. This solution was maintained at 37 °C under magnetic stirring. At each time point, 1 ml of the solution was taken out and centrifuged at 3000 RCF for 5 min to remove all non-dissolved solid components. The concentration of the RhB in the collected supernatant was analyzed using UV-visible spectrophotometer at 553 nm using the methods described earlier. To determine release due to HIFU, samples were exposed to 10 min of 1.1 MHz HIFU at 10% duty cycle and 3.9 MPa peak negative pressure amplitude. Experiments were performed in triplicate.

### *In vitro* evaluation of RhB-mcPLGA MPs penetration and RhB release

Penetration depth and release rates from HIFU-guided implantation of RhB-mcPLGA MPs were measured in an *in vitro* agarose tissue model. The agarose phantom was made following the procedure mentioned above. The location of the HIFU focus was also conducted following the methods described above for acoustic characterization. After ultrasound exposure at 1.1 MHz center frequency, 3.9 MPa peak negative pressure, and 10% duty cycle, the flow channel was washed with DI water to remove the remaining RhB-mcPLGA MPs. The agarose phantoms were cut to 20 × 10 × 10 mm cubes and were placed on a glass slide to measure the penetration using a microcope. The excised agarose phantoms were transferred to a sealed petri dish, and incubated with PBS at 37 °C. At each time point, the agarose phantoms were evaluated with a fluorescence microscope (Axio Observer Z1 inverted widefield, Zeiss) to evaluate the release of RhB from mcPLGA MPs. For the quantitative analysis of fluorescence, we used a ZEISS Microscope Software ZEN 2.3 lite (blue edition) to measure the total fluorescence of a fixed region (50 × 200 µm) for 3 independent agarose phantoms across 15 days. Release was defined as fluorescence decay at same exposure conditions across 15 days.

### HIFU-Guided implantation of RhB-mcPLGA and RhB/DAPI-mcPLGA MPs in an *ex vivo* porcine artery

The connective tissue of the pig artery (adventitia) was first stripped off. The artery was then cleaned by passing water through the lumen. After, it was cut into two 5 cm length portions. The artery was placed and secured into the sample flow chamber. Prepared agarose solution (as described earlier) was then poured into the chamber at a temperature of 45 to 50 °C. The entire setup was cooled to room temperature under ambient conditions.

Among the two arteries, one remained as a control whilst the other was exposed to HIFU. A syringe pump passed RhB-mcPLGA MPs at a concentration of 1 mg/ml through the lumen of the test artery at a rate of 0.2 ml/min. The other end of the artery had tubing that led to a collection beaker. The artery chamber was then placed in the water tank and the focus of the HIFU transducer was aligned to the backwall of the artery. The HIFU exposure conditions were as follows: 1.1 MHz center frequency, 3.9 MPa peak negative pressure, 2 Hz PRF, and 10% duty cycle. After the ultrasound exposure, the chamber was taken out and the section of the artery exposed to ultrasound was removed. A similarly sized section from the control artery was also removed. The artery fragments were then cryostat sectioned for further analysis. To do so, each artery fragment was cut into thin slices. Each thin slice was then frozen with the O.C.T. compound onto the specialized metal grids that fit onto the cryostat (CM1950; Leica Cryostat, Germany). Then the metal grid was mounted into the cutting chamber and the arteries were sectioned and transferred to glass microscope slides. The sectioned arteries on the slides were DAPI stained following a conventional protocol. In short, the sectioned slides were rinsed with filtered deionized water with excess water dried off. DAPI was added to the slide and allowed to rest for three to five minutes. After, a coverslip was added. The slides were imaged using a confocal laser scanning microscope (Zeiss LSM 800).

### Release of DAPI from mcPLGA-MPs in porcine artery

To determine the distribution of the payload released from the degradation of mcPLGA-MPs delivered to the sub-endothelial region of *ex vivo* porcine artery, the artery was implanted with RhB and DAPI loaded mcPLGA MPs (RhB/DAPI-mcPLGA MPs) with HIFU using the same parameters as the HIFU-guided implantation of RhB-mcPLGA MPs study (1.1 MHz center frequency, 3.9 MPa peak negative pressure, 2 Hz PRF, and 10% duty cycle). Post ultrasound exposure, the section of the artery at the focus of the HIFU transducer was removed. A similarly sized section from a control artery was also removed. Both were placed in PBS at 37 °C. At each time point, the artery fragments were cryostat sectioned and imaged using a confocal laser scanning microscope (Zeiss LSM 800).

## Supplementary information

Supporting Information
