# Author Correction: Remote targeted implantation of sound-sensitive biodegradable multi-cavity microparticles with focused ultrasound

**DOI:** 10.1038/s41598-020-66546-0

**Published:** 2020-06-04

**Authors:** Xiaoqian Su, Reju George Thomas, Lakshmi Deepika Bharatula, James J. Kwan

**Affiliations:** School of Chemical and Biomedical Engineering, Nanyang Technological University, Singapore, 637459 Singapore

Correction to: *Scientific Reports* 10.1038/s41598-019-46022-0, published online 03 July 2019

This Article contains typographical errors in the Acknowledgements section.

“This research is supported by the Singapore Ministry of Health’s National Medical Research Council under its NMRC/OFYIRG/NOV006/2016.”

should read:

“This research is supported by the Singapore Ministry of Health’s National Medical Research Council under its NMRC/OFYIRG/0034/2017.”

