# Supplementary material for: Remote targeted implantation of sound-sensitive biodegradable multi-cavity microparticles with focused ultrasound
Source: Sci Rep. 2019 Jul 3;9:9612. doi: 10.1038/s41598-019-46022-0 (PMC6610131; doi:10.1038/s41598-019-46022-0)
Supplement: Supplementary file 1 — Supporting Information [file 41598_2019_46022_MOESM1_ESM.pdf]

Supporting Information

Remote targeted implantation of sound-sensitive biodegradable multi-cavity microparticles with focused ultrasound

Xiaoqian Su[a], Reju George Thomas[a], Lakshmi Deepika Bharatula[a], and James J. Kwan[a,*]

[a]*School of Chemical and Biomedical Engineering, Nanyang Technological University, Singapore, 637459*

*Corresponding author: E-mail: jameskwan@ntu.edu.sg

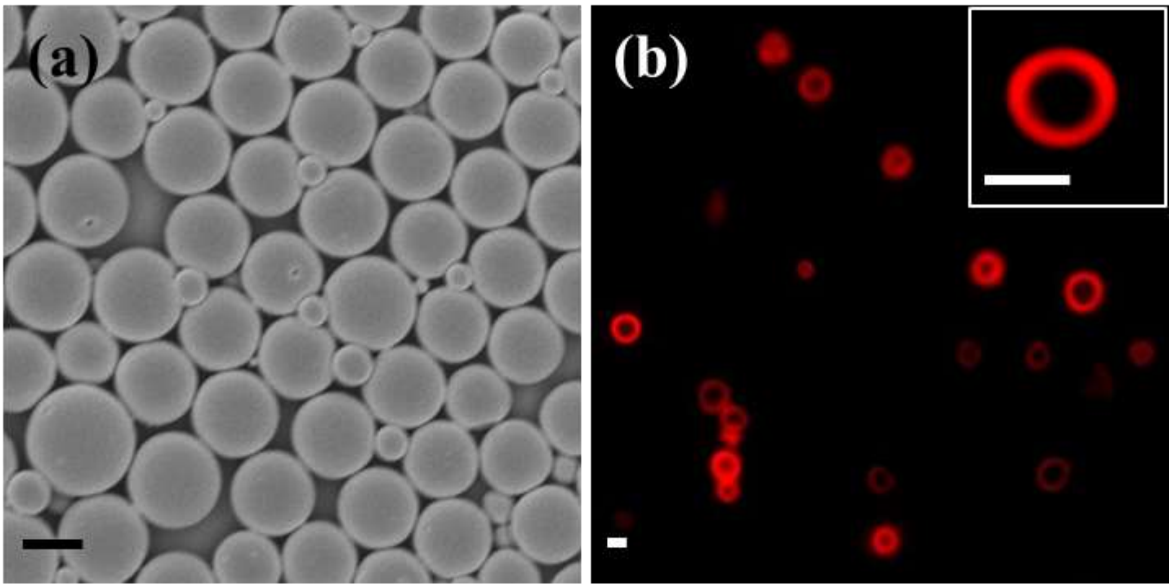

Figure S1. (a) SEM and **(b)** fluorescence images of RhB-hsPLGA MPs. The scale bars represent 1 μm.

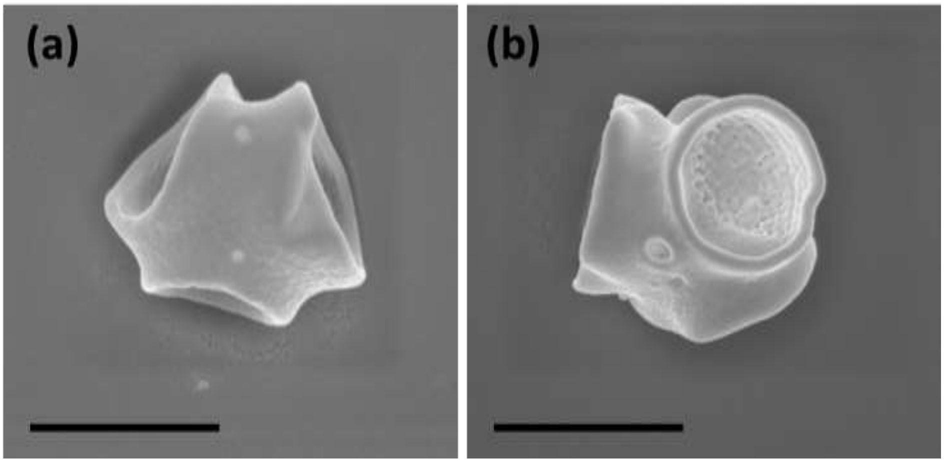

Figure S2. (a) Side and **(b)** bottom view of SEM images of RhB-mcPLGA MPs**.** The scale bars represent 1 μm.

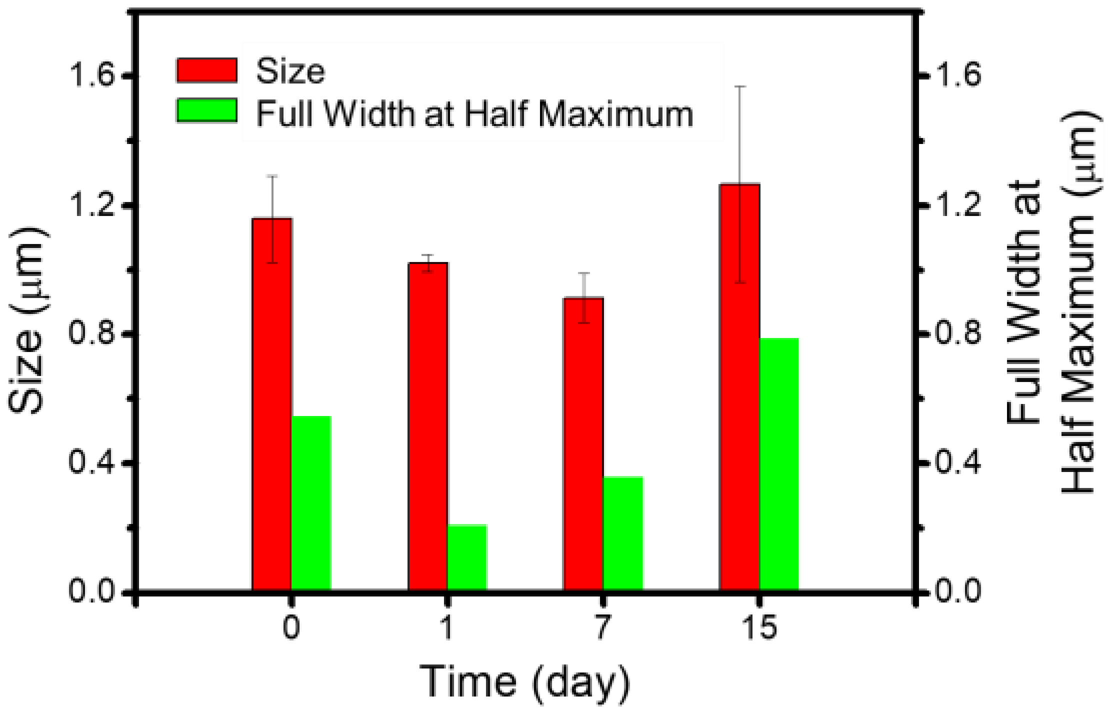

Figure S3. Average size and full width at half maximum of the size distribution of RhB-mcPLGA MPs incubated in PBS at 37°C across 15 days.

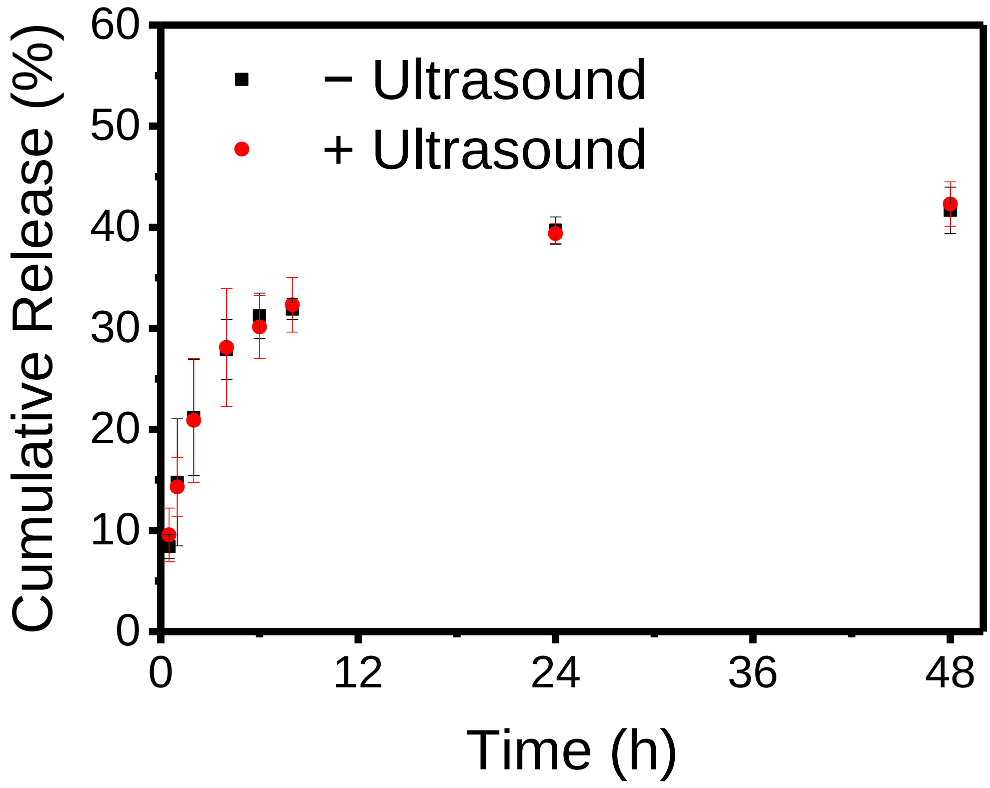

Figure S4. Model drug RhB release profile from RhB-mcPLGAs MPs before and after 10 min HIFU exposure (1.1 MHz center frequency, 10% duty cycle, 2 Hz pulse repetition frequency).

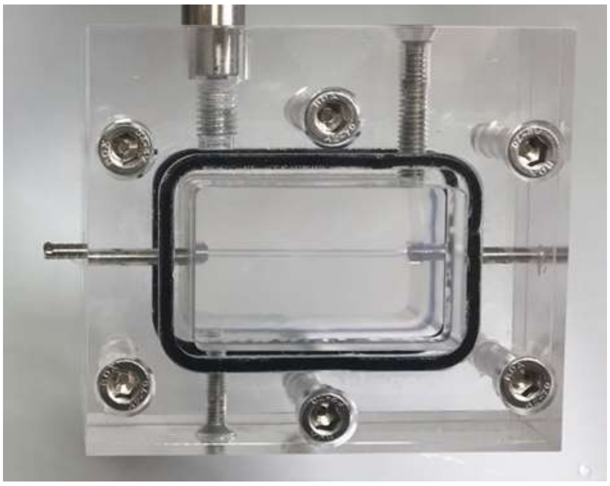

Figure S5. Image of the agarose chamber with a 1.6 mm flow channel.

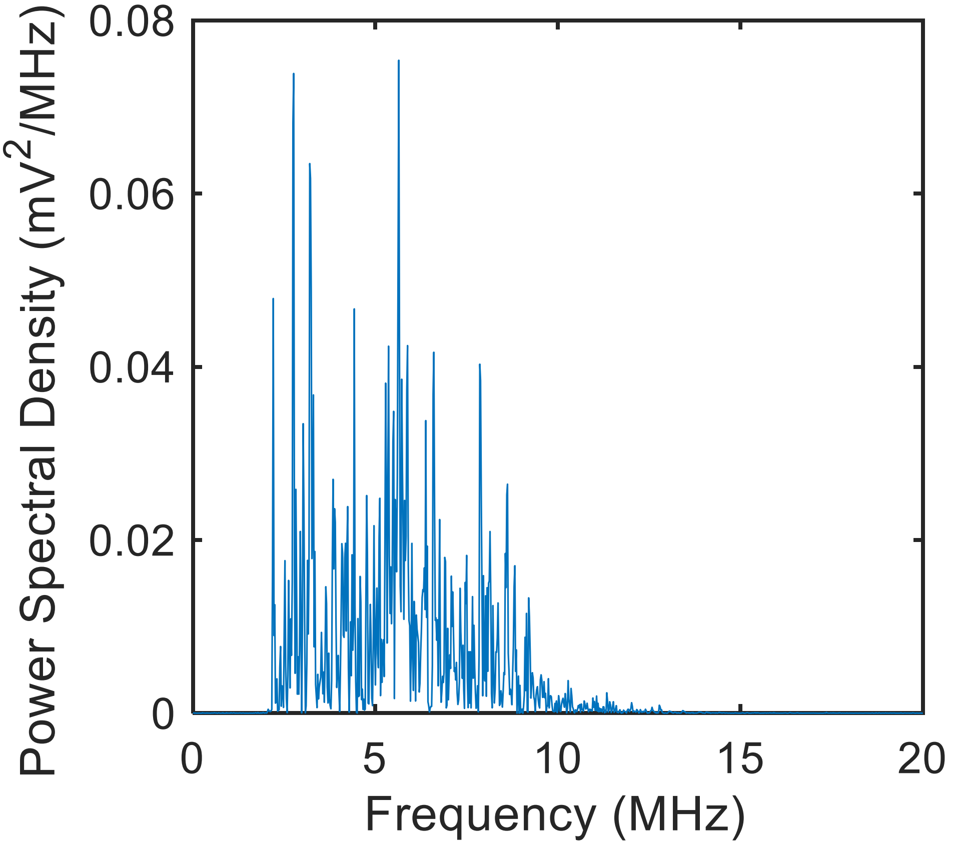

Figure S6. Representative power spectrum from RhB-mcPLGA MPs after exposure to HIFU.

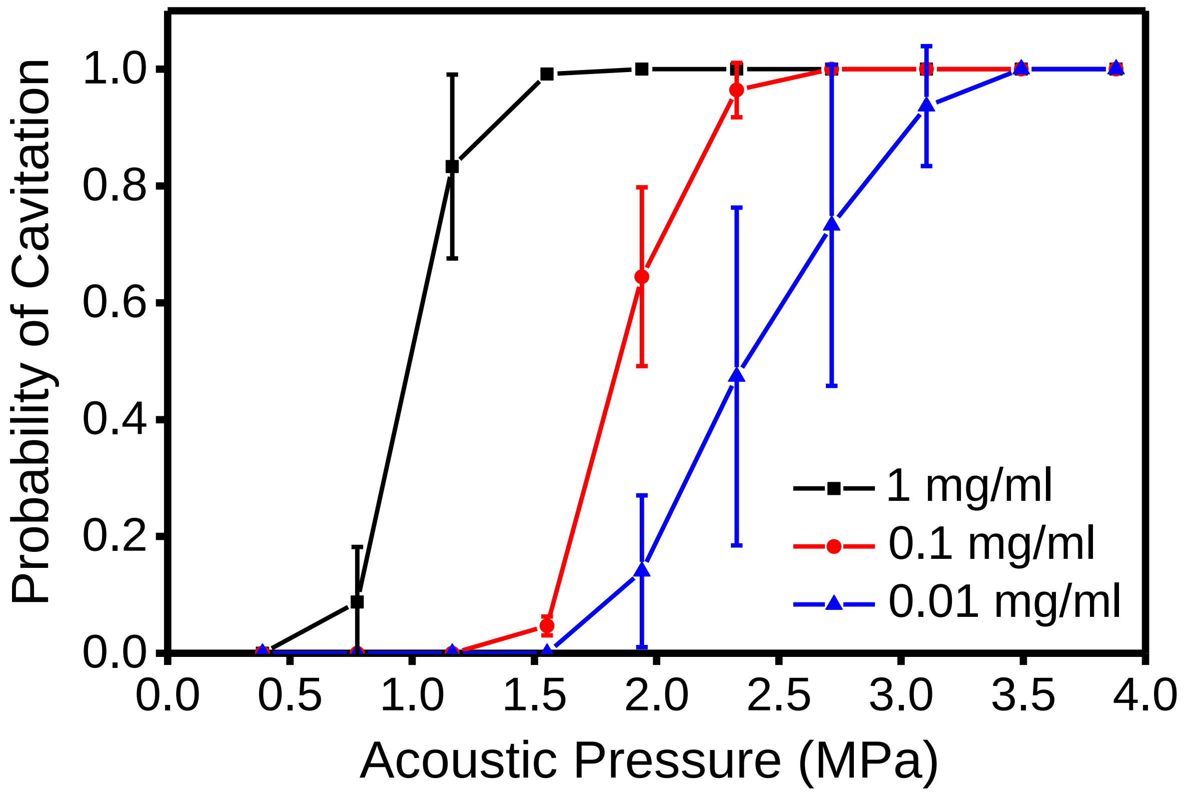

Figure S7. Probability of cavitation of RhB-mcPLGA MPs with a concentration of 1, 0.1, and 0.01 mg/ml exposed to HIFU (1.1 MHz center frequency).

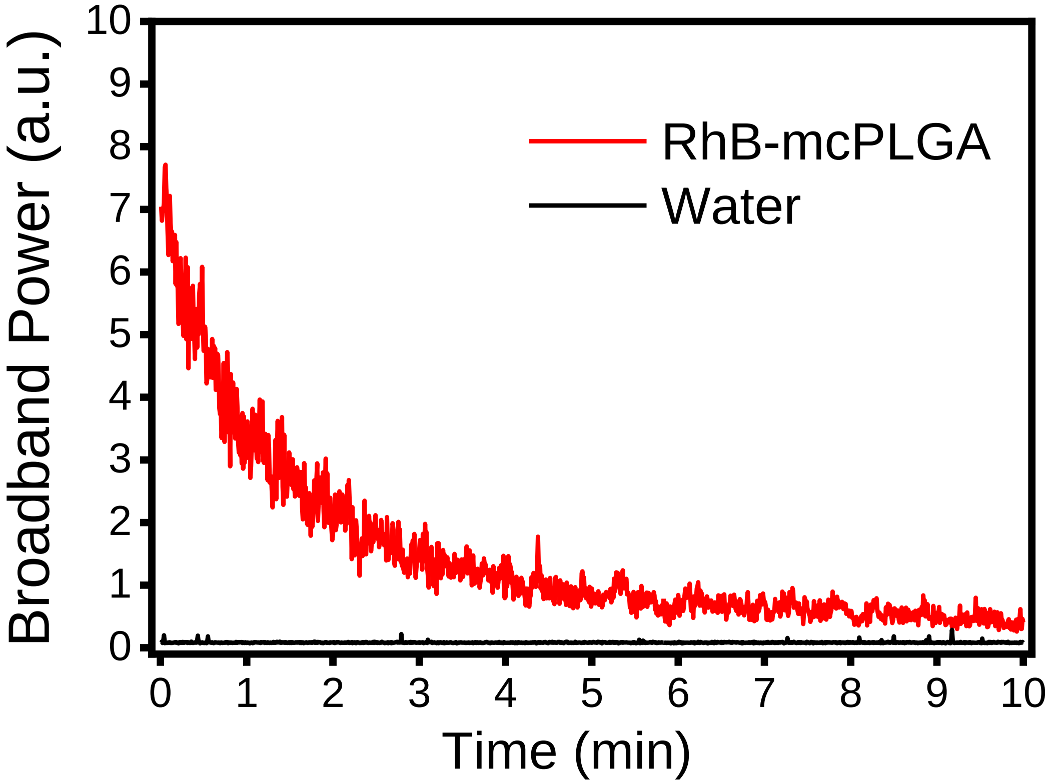

Figure S8. Broadband power of RhB-mcPLGA MPs across 10 min of ultrasound exposure.

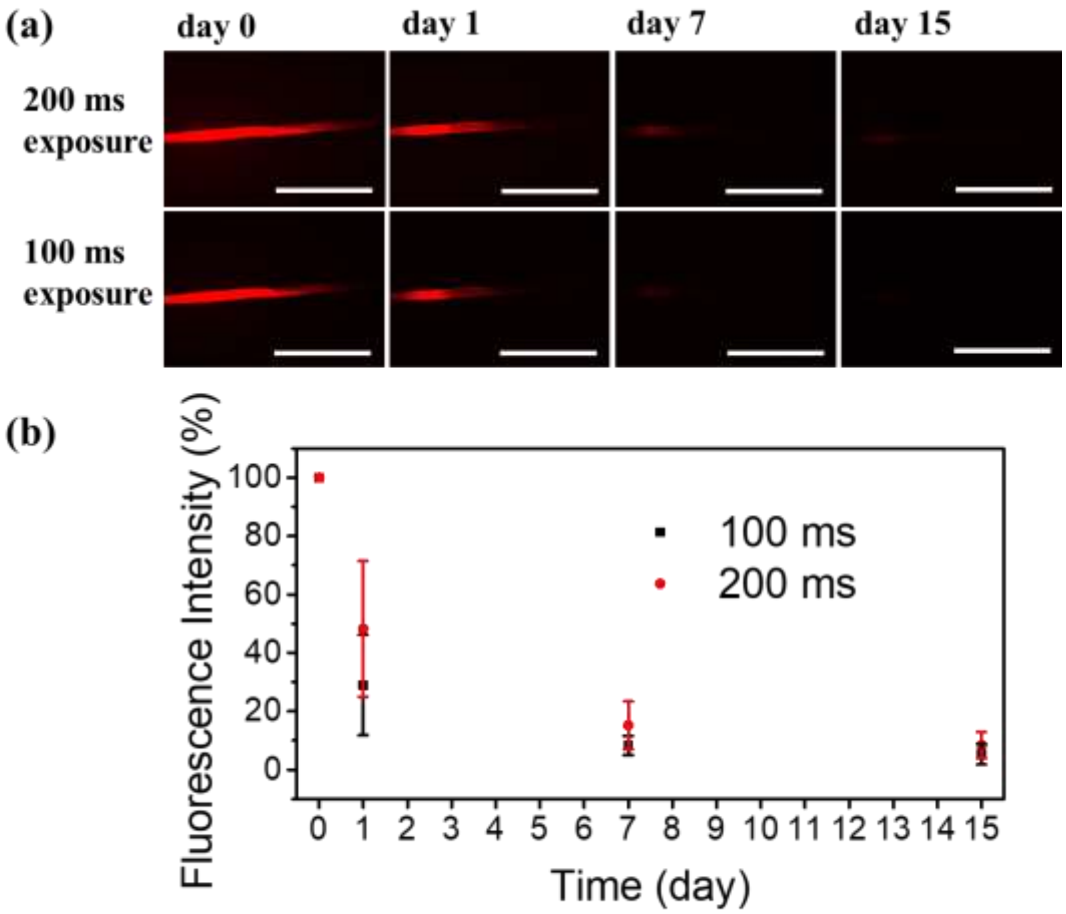

Figure S9. (a) Representative fluorescent images of the RhB-mcPLGA MPs embedded in agarose phantoms at exposure time of 200 and 100 ms, respectively**. (b)** Quantitive analysis of change in fluorescence intensity across 15 days with same ROIs (n=3 independent samples). The scale bars represent 500 μm.

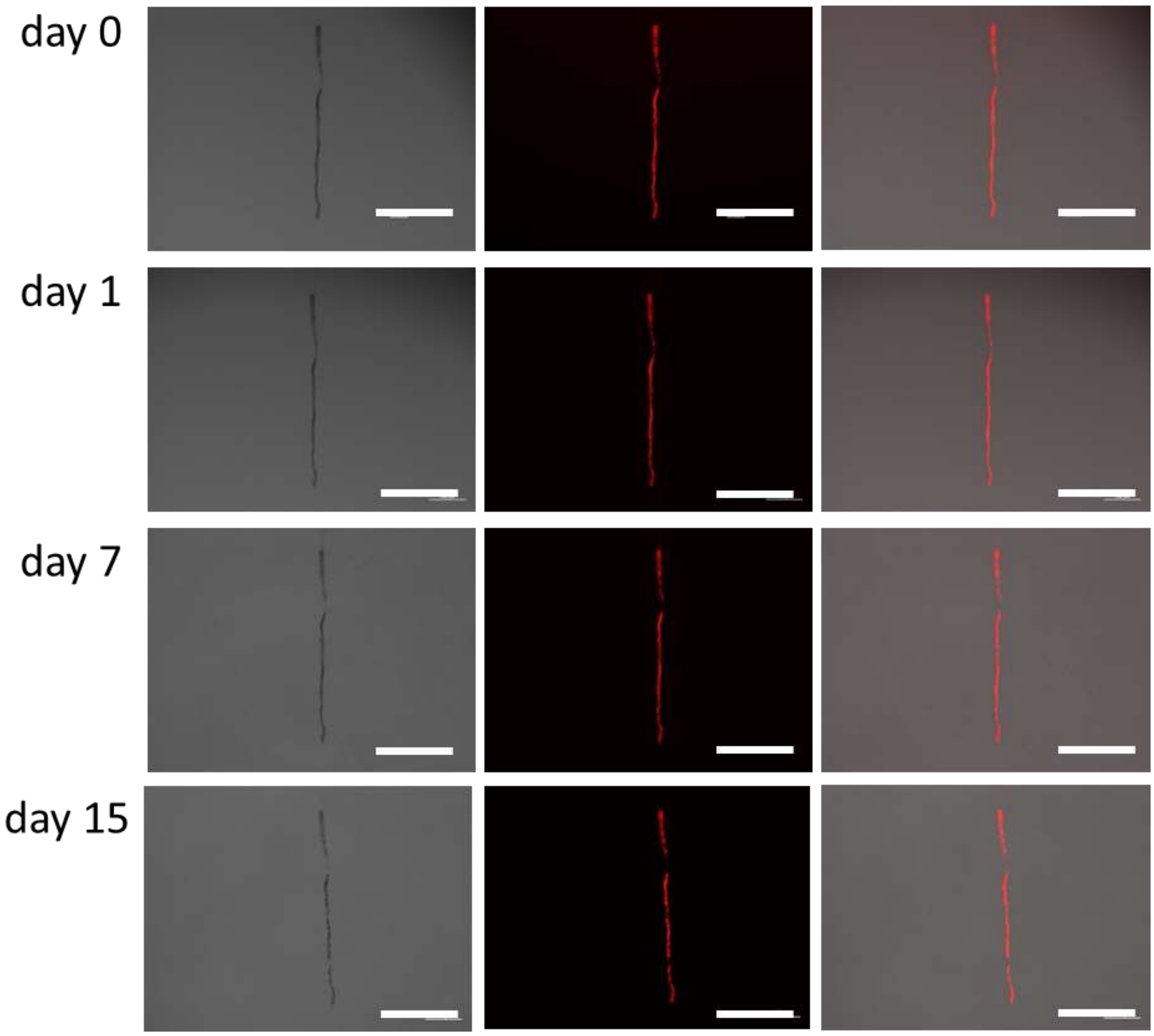

Figure S10. Fluorescence images of RhB-mcPLGA MPs embedded in tissue-mimicking agarose phantoms incubated at 4°C across 15 days. The scale bars represent 400 μm.

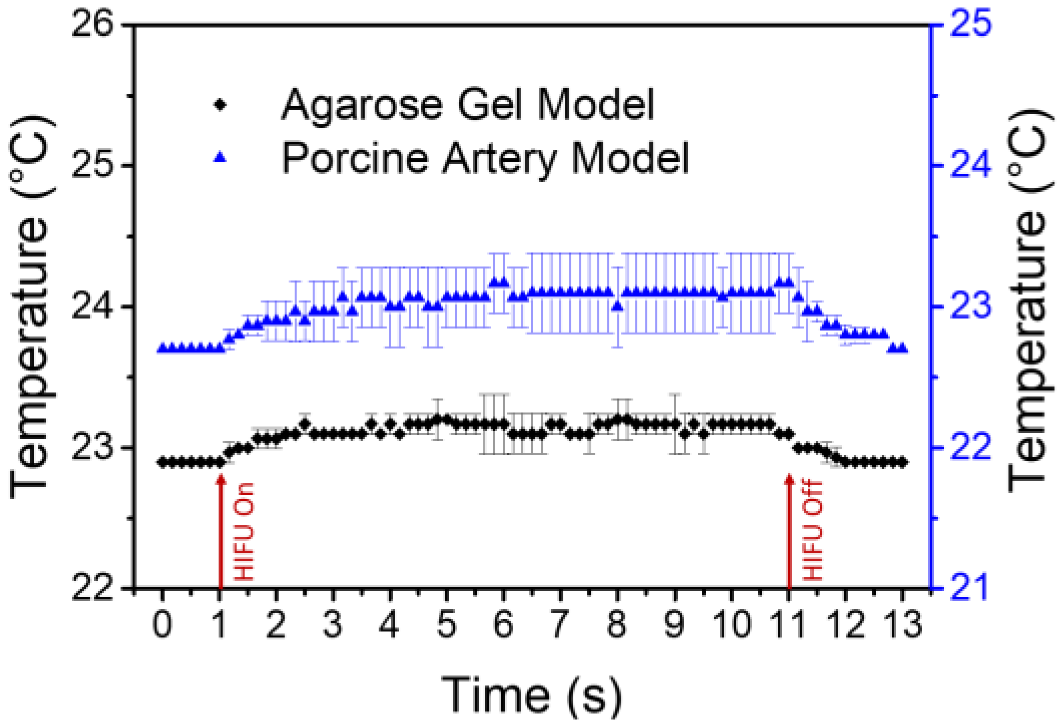

Figure S11. Temperature response in both agarose and artery model for 10 min of ultrasound exposure. Error bars represent the standard deviation of three independent samples.

S11 Section 1. Methods

The agarose phantom and artery phantom was made using the method described in the main manuscript. To determine the thermal effect due to HIFU, we inserted a digital thermocouple probe (BlueTherm Duo, Thermoworks, UK) next to the lumen of both phantoms at the focus of the HIFU. We then exposed the vessels to ultrasound (10% duty cycle, 3.9 MPa, 2Hz PRF, 10 min). Temperatures were monitored every 10 s before (1 min), during (10 min), and post (2 min) exposure to HIFU.

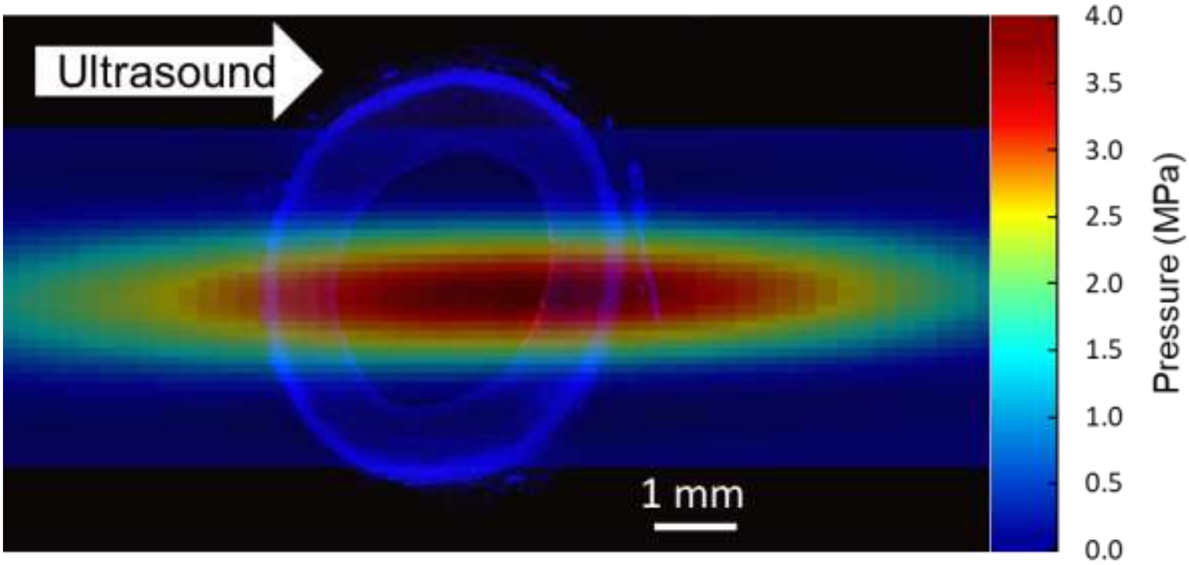

Figure S12. Fluorescent image of a porcine artery after exposure to HIFU and RhB-mcPLGA MPs with an overlap of the normalized acoustic pressure map of the beam formed by the 1.1 MHz HIFU transducer (scale is 1:1). The arrow shows the direction of ultrasound.
